# Predicted Future Mortality Attributed to Increases in Temperature and PM_10_ Concentration under Representative Concentration Pathway Scenarios

**DOI:** 10.3390/ijerph17072600

**Published:** 2020-04-10

**Authors:** Jiyun Jung, Jae Young Lee, Hyewon Lee, Ho Kim

**Affiliations:** 1 Graduate School of Public Health, Seoul National University, 1 Gwanak-Ro, Gwanak-Gu, Seoul 08826, Korea; bestjudy@hanmail.net; 2 Institute of Health and Environment, Seoul National University, 1 Gwanak-Ro, Gwanak-Gu, Seoul 08826, Korea; jaeyoung.lee@alumni.stanford.edu (J.Y.L.); woniggo@gmail.com (H.L.); 3 Department of Neuropsychiatry, Seoul National University Bundang Hospital, Seongnam 13620, Korea

**Keywords:** modification effect, high-temperature, inhalable particulate matter, regional variation

## Abstract

As climate change progresses, understanding the impact on human health associated with the temperature and air pollutants has been paramount. However, the predicted effect on temperature associated with particulate matter (PM_10_) is not well understood due to the difficulty in predicting the local and regional PM_10_. We compared temperature-attributable mortality for the baseline (2003–2012), 2030s (2026–2035), 2050s (2046–2055), and 2080s (2076–2085) based on a distributed lag non-linear model by simultaneously considering assumed levels of PM_10_ on historical and projected temperatures under representative concentration pathway (RCP) scenarios. The considered projected PM_10_ concentrations of 35, 50, 65, 80, and 95 μg/m^3^ were based on historical concentration quantiles. Our findings confirmed greater temperature-attributable risks at PM_10_ concentrations above 65 μg/m^3^ due to the modification effect of the pollutants on temperature. In addition, this association between temperature and PM_10_ was higher under RCP8.5 than RCP4.5. We also confirmed regional heterogeneity in temperature-attributable deaths by considering PM_10_ concentrations in South Korea with higher risks in heavily populated areas. These results demonstrated that the modification association of air pollutants on health burdens attributable to increasing temperatures should be considered by researchers and policy makers.

## 1. Introduction

In recent years, health risks have increased due to climate change associated with advancing industrialization. Studies based on historical data have shown the impact of temperature on human health, including the total and cause-specific mortality [1] and morbidity [2]. In particular, increased mortality was associated with extremely cold or hot days, usually defined as percentile and value of temperature [1,3,4].

Due to adverse effect of climate change on human health, the projection of future mortality rates under representative concentration pathway (RCP) scenarios has become an important area of study. RCP is based on different energy use, economic, and demographic assumptions, which is the typical trajectory of gas emissions recommended by the 5th Intergovernmental Panel on Climate Change (IPCC) assessment report. RCP8.5 is supposed to be the current trend of GHG emissions as Business As Usual (BAU), while RCP4.5 is a mitigation scenario. In the 51 largest cities of China, the projected heat-related excess mortality in 2041–2061 relative to 1970–2000 was 37,800 and 31,700 per year under RCP8.5 and RCP4.5, respectively [5]. A study of metropolitan areas in the United States also estimated heat-related deaths under RCP8.5 and RCP4.5 during 2085–2095 compared to 1992–2002, highlighting the risk of high temperatures [6]. Likewise, the projected heat-related risks on health have been studied in England [7], India [8], and South Korea [9]. However, few studies have considered the modification effect of air pollution on temperature-related mortality.

The complex interactions between temperature and air pollutants have been investigated based on historical data and have shown an increased risk of mortality due to the modification effect [10,11,12]. Two methods have been used to investigate the interaction of air pollution and temperature: (1) the modification effect of air pollution on temperature-related mortality, and (2) the modification effect of temperature on air-pollutant-related mortality. Using the former method, we estimated the modification effect of air pollution on temperature-related mortality. The effect of air pollution on temperature-related mortality has been identified in several studies [13,14,15]. However, few projection studies have examined effects on health by considering both particulate matter (PM) and temperature because of the difficulties in projecting future PM levels. Predicting the effect of climate change on PM is challenging because of unreliable deterministic predictive variables such as precipitation, frequency, and mixing depth [16].

Therefore, to better understand the modification effect of air pollution on temperature-attributable mortality, our research analyzed the effects on health by simultaneously considering not only the assumed levels of PM_10_, but also the projected temperature under RCP scenarios. Unlike the data for PM_2.5_ concentrations, PM_10_ was measured across the country. In this study, the assumed PM_10_ level was expressed in a simple scenario based on historical PM_10_ concentration quantiles. The current and anticipated health effects of temperature were considered by including the modification effect of PM_10_. The results of our study could be used to provide guidelines for policy makers on hazardous air pollution levels under all climate change scenarios.

## 2. Methods

### 2.1. Data Collection

We obtained historical data on the baseline (2003–2012) and projected data for the projection period (2030s, 2050s, and 2080s) from 229 districts in South Korea. For baseline, we collected non-accidental mortality from the Korean Statistical Information Service (KOSIS) and historical climate data, such as daily mean temperature, the ambient daily concentration of PM_10_, and humidity, from the Korean Meteorological Administration (KMA) for 229 districts in South Korea. We obtained historical PM_10_ measured from 274 stations through the national atmospheric monitoring network. We assigned the average of hourly basis into daily mean concentration. For the projection period, future daily mean temperature under representative concentration pathways (RCPs) scenarios were obtained from the Climate Change Information Center. RCP scenarios were simulated for up to 2100 by the Hadley Centre Global Environmental Model version 3 (HadGEM3-RA, Korea) regional climate model and the Modified Korean-Parameter-elevation Regressions an Independent Slopes Model (MK-PRISM, Korea) in Korea. Our study employed RCP4.5 and RCP8.5 for the 2030s (2026–2035), 2050s (2046–2055), and 2080s (2076–2085) from 230 districts. Because the future climate data were provided for 230 districts, the average temperature of two districts in the predicted period, namely Chungcheongbuk-do Cheongwon-gun and Chungcheongnam-do Yeonggi-gun, was used for the Sejong-si district in historical data to match the administrative district.

### 2.2. Temperature-Mortality Relationship Considering Interaction between PM_10_ and Temperature

City-specific exposure-response curves of seven major cities in Korea were extracted using a distributed lag non-linear model (DLNM) with the temperature, day of week, time, and humidity. In addition, the interactions between temperature and PM_10_ were included or not included, depending on the analysis. The DLNM method adjusted for the non-linear relationship and the lag effect between temperature and non-accidental mortality [17]. A quadratic B-spline function with knots placed at the 10th, 75th, and 90th percentiles was selected for assessing the temperature-mortality relationship. We placed lag knots at intervals of the log scale. Maximum lags of 21 were used with three degrees of freedom because cold-related mortality has the highest association with longer lag [18,19], and we used time adjustment by using a natural spline (ns) with eight degrees of freedom based on QAIC. The complete regression model used in Equation (1) is given by

(1) $$\log\left(E\left(Y\right)\right)=\alpha +cb\left(temp\right)+cb\left(temp\right)*cPM_{10}+PM_{10}+Factor\left(day\;of\;week\right)\;+ns\left(date,\;df=8*years\right)+humidity$$

where cPM_10_ is the centered PM_10_ at 35, 50, 65, 80, or 95 $\mu g/m^{3}$. *PM*_10_ concentration at baseline was used in the model without removing outliers. We used humidity as the covariate because increased humidity was associated with decreased temperature as well as formation of secondary particles. *cPM*_10_ indicated the modeled *PM*_10_ concentration, which was centered at *PM*_10_ for 35, 50, 65, 80, or 95 μg/m^3^. In addition, Y, α, and cb(temp) indicated the death counts, the intercept, and the cross-basis object for temperature, respectively.

The estimated association between temperature and mortality in seven major cities was then pooled through multivariate meta-regression models using the coefficients of our basic model. We included the difference in the temperature range and average temperature of seven cities as meta-regression predictors to consider district heterogeneity [20] and to obtain stable relative risk. The pooled estimate from the seven cities was used for all other districts. The relative risks (RR) were based on 99th versus 95th percentile temperatures for 229 districts and 4 periods (baseline and projected periods) in order to investigate extreme heat effects, assuming that adaptation has occurred in the projected period [21]. Therefore, we obtained different RRs by district and period because the 95th and 99th percentile temperatures varied by district and period, even though we derived one curve for the relationship between mortality and temperature in Korea.

### 2.3. Historical Mortality at Baseline and Estimated Mortality of Projected Period

The number of deaths at baseline was presented as the sum of non-accident-related mortality between 2003 and 2012. Future mortality was estimated based on the United Nations (UN) projection from the World Population Prospects (WPP 2017) medium-variant scenario. The medium-variant scenario was selected for this work because our research focused only on the modification effect of PM_10_ on temperature rather than on the trends of deaths that depend on various mortality scenarios. Thus, our use of the medium-variant scenario reduced the uncertainty in our estimation of the number of future attributable deaths. Estimates from the WPP model are given in five-year increments, so the estimated deaths for the predicted period in the 2030s, 2050s, and 2080s were average estimates between 2025–2030 and 2030–2035, 2045–2050 and 2050–2055, and 2075–2080 and 2080–2085, respectively. 

Because the UN mortality data provided only the total number of deaths in Korea, the mortality rate of the baseline period (the number of deaths per district divided by the total number of deaths) was used to predict the mortality in 229 districts. Therefore, the ten-year average deaths of the 229 regions in the projected period were calculated in Equation (2) using the mortality rate for the ten-year average deaths for those regions in baseline:

(2) $$Deaths_{ij}=predicted\;deaths_{i}\times mortality\;rate\;of\;baseline_{j}$$

(i=1,2,3 and j=1,…,229)

(3) $$Attributable\;deaths_{ij}=\frac{RR_{ij}-1}{RR_{ij}}\times Deaths_{ij}$$

Here, *i* and *j* indicate the projected period (2030s, 2050, and 2080s) and the district number for the 229 considered districts, respectively. Finally, Equation (3) estimates attributable deaths. All analyses were conducted by R version 3.1.0 (R Foundation for Statistical Computing, Vienna, Austria).

## 3. Results

Table 1 and Figure S1 show the 95th and 99th percentile temperature during baseline and the projection period (2030s, 2050s and 2080s). The 95th and 99th percentile temperatures under RCP8.5 were both higher than those under RCP4.5. Both percentiles in the 2080s were higher than those in the 2030s.

**Figure 1:**
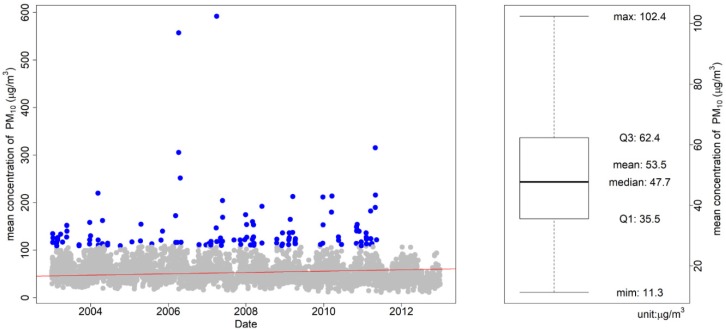
Mean PM_10_ concentration distribution (grey dot) with outlier (blue dot) and its trend (red line) during the baseline period (2003–2012) (**left**), and the quartile concentration of the baseline period without outliers, which ranged from Q1 − 1.5 × IQR to Q3 + 1.5 × IQR (**right**).

Figure 1 shows the mean PM_10_ concentration based on historical data from 2003 to 2012. The mean and standard deviation of the baseline were 53.61 μg/m^3^ and 28.48 μg/m^3^, respectively. Outliers, which were defined as values outside the lower limit (Q1 − 1.5 × interquartile range (IQR: Q3–Q1)) and upper limit (Q3 + 1.5 × IQR), were removed to identify the trends over a decade. The mean and standard deviation after removing outliers were 50.5 μg/m^3^ and 20.03 μg/m^3^, respectively. Because PM_10_ concentration trends in the baseline were not evident, the 25th, 50th, and 75th percentiles of the outlier-removed data were maintained in the projected period. Therefore, we utilized the assumed future PM_10_ concentrations of 35, 50, and 65. The concentrations of 80 and 95 μg/m^3^ were additionally analyzed based on the interval of 25th, 50th, and 75th percentiles to verify the effect on relatively high concentrations. 

**Figure 2:**
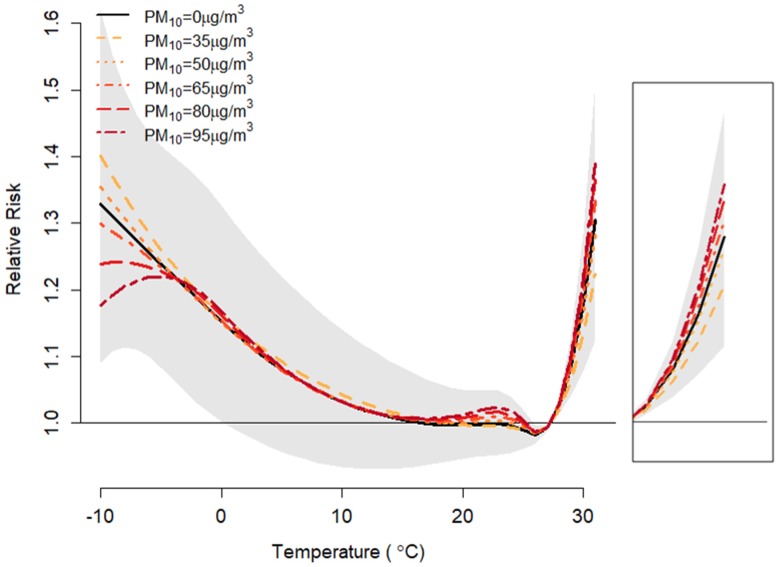
Estimated temperature-mortality curves with various PM_10_ concentrations (0, 35, 50, 65, 80, and 95 μg/m^3^) in South Korea (**left**) with 95% confidence interval (gray shaded region), and enlarged high-temperature region (**right**).

The relationships between temperature and mortality were non-linear for all considered PM_10_ concentration values (Figure 2). Similar associations were found in seven major cities before meta-analysis (Figure S2). The relative risk shown here is the temperature-attributable risk for various PM_10_ levels. As shown in Figure 2, the relative risk for PM_10_ concentrations of 35 and 50 μg/m^3^ was lower than that not considering the PM_10_ concentration (0 μg/m^3^). However, when the concentration of PM_10_ varied from 65 μg/m^3^ to 95 μg/m^3^, the relative risk increased. Thus, as PM_10_ concentration increased above 50 μg/m^3^, the relative risks of high temperature on mortality also increased. In addition, we found the tendency to decrease relative risk as the lag period get longer (Figure S3).

**Figure 3:**
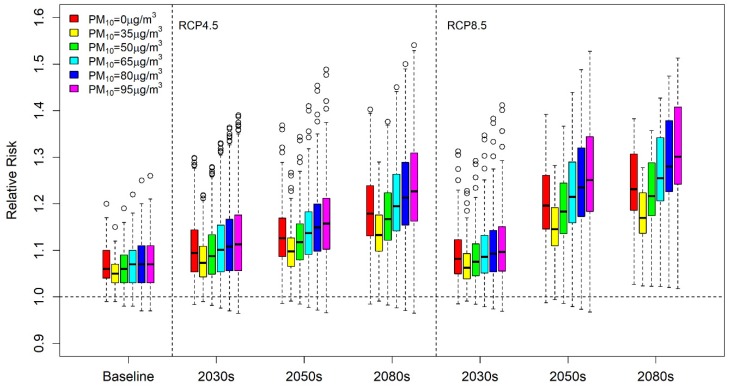
Relative risk boxplots of the 229 districts for the baseline and projection periods (2030s (2026–2035), 2050s (2046–2055), and 2080s (2076–2085)) under predicted temperature scenarios (RCP4.5 and 8.5) and the medium-variant population scenario. Red, yellow, green, light blue, navy, and pink colors correspond to PM_10_ levels of 0, 35, 50, 65, 80, and 95 μg/m^3^, respectively.

Figure 3 shows boxplots for the relative risk in the baseline, 2030s, 2050s, and 2080s when PM_10_ concentration increases. Table S1 also shows the mean temperature-attributable relative risks and 95% confidence interval with various PM_10_ levels in the 229 districts in South Korea. In the baseline period, the average relative risk without considering PM_10_ concentrations was equal to or lower than those between 50 μg/m^3^ and 65 μg/m^3^. Interestingly, risks at relatively low concentrations such as 35 μg/m^3^ and 50 μg/m^3^ were lower than those not considering the PM_10_ interaction effect. However, the relative risk of temperature, considering the association of temperature and air pollution under all scenarios and PM_10_ concentrations, tended to increase with concentrations of PM_10_ at higher temperature ranges. Table 2 and Figure S4 show the mean of the attributable death counts for the baseline and projected period by applying the relative risk and estimated mortality for 229 districts. Generally, attributable deaths increased with the period and scenario. When the PM_10_ concentration was assumed to be 95 μg/m^3^, the temperature-attributable mortality increased from 74.89 to 552.62 under RCP4.5, and to 673.91 under RCP8.5. The results indicated that PM_10_ concentrations affect temperature and contribute to increased temperature-attributable deaths. Since RCP8.5 assumes greater temperature increases than RCP4.5, the number of attributable deaths was greater under RCP8.5 for both the 2050s and 2080s. However, this tendency was not observed in the 2030s because the projected temperatures under RCP4.5 and 8.5 were similar.

**Figure 4:**
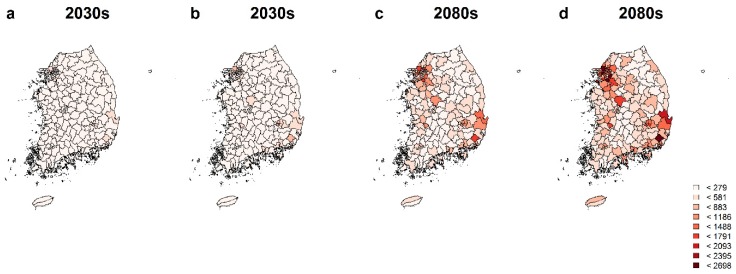
National distribution of temperature-attributable deaths considering simultaneous effects of PM_10_ and temperature when PM_10_ was assumed to be 35 μg/m^3^, for (**a**) and (**c**), and 95 μg/m^3^, for (**b**) and (**d**), under RCP8.5 in the 2030s and 2080s.

In this study, we also confirmed regional heterogeneity in temperature-attributable deaths considering the PM_10_ concentrations in South Korea because different relative risks and deaths were applied in attributable deaths. As shown in Figure 4, metropolitan cities such as Seoul and Busan with higher population densities showed higher attributable mortality than other locations. In addition, the number of attributable deaths depended on the characteristics of various districts such as temperature and estimated mortality.

## 4. Discussion

This study examined how future temperature-related mortality changes under different assumed PM_10_ concentrations. We observed a modification effect due to PM_10_ in the relationship between temperature and mortality over the predicted periods, and we identified higher mortality risks at ranges of PM_10_ concentration over 65 μg/m^3^. Our results that confirmed the modification effect at the baseline agreed with previous studies conducted by Chen et al. (2018) [22] and Li et al. (2015) [23]. 

In this study, the attributable deaths in both RCP scenarios increased along the period because of temperature increase. In addition, the estimated temperature-attributable deaths at PM_10_ concentrations of 65 and 95 μg/m^3^ were higher than those at concentrations of 0, 35, and 50 μg/m^3^. This result suggests that the interaction effect between temperature and PM_10_ may depend on the PM_10_ concentration. Surface and atmospheric cooling occurs when aerosols are reflected, while atmospheric warming was observed when absorbing aerosols. However, various properties of aerosol and characteristics of surfaces could make the effects of atmospheric aerosols on temperature different [24]. Although the underlying mechanism of how air pollution modifies the temperature effect is unclear, several hypotheses have been proposed. First, there may be a synergistic effect between the ambient temperature and air pollution on mortality [10]. Correlation between those two variables is generally high in many places [25], and the PM concentration is highest during the warm season [26]. Second, air pollutant exposure may increase because people tend to open doors and windows or go outside on a warmer day [22]. Moreover, air pollutants are more readily absorbed by the body when temperature is high due to increased skin permeability and increased respiratory rate [23]. Third, a biological mechanism supporting the modification effect of PM_10_ on temperature is plausible, especially in the case of cardiorespiratory disease, which can lead to mortality through inhalation [14], and modulation of the automatic nervous system [27]. Further, temperature could aggravate preexisting disease because of physiological and psychological stress [27]. Therefore, the results of our study suggest the importance of ambient PM_10_ emission control because of the greater temperature-attributable risks at concentrations above 65 μg/m^3^ due to the modification effect.

Our results also show regional variations, which demonstrated greater health risks in metropolitan areas. Local variation of health risk caused by temperature can be manifested by various sources. Region-specific temperatures may be strongly affected by orographic precipitation, coastlines, and other local climate patterns [28]. In addition, health risks depend on social, economic, and demographic characteristics [29]. In this study, the attributable risk was estimated to be greater in metropolitan areas with higher mortality; however, determinants of health risks between rural and metropolitan vary [30]. The heat island effect caused by poor ventilation, increased thermal storage in the urban environment, and heat generated from air conditioning and vehicles may increase risks in metropolitan areas [31]. Moreover, people in high density settlements are more vulnerable to higher temperatures and thermal discomfort [32].

There were several limitations on our study. First, we assumed that the 229 district-specific mortality rates in the projected period did not change from the current regional mortality rates because of difficulty in estimating the changing rates. We assumed current association between PM_10_ and temperature is maintained in future periods, not considering adaptation and mitigation of climate change and local particulate matter. However, regional mortality differences could be caused by a variety of reasons [33], and rural regions can be burdened by diseases that are not prevalent in metropolitan areas [34]. Second, cold-related mortality with PM_10_ modification was not addressed in this study. Based on Figure 2, the tendency of cold temperature-attributable deaths for various PM_10_ levels will differ from that of high temperature. Therefore, further studies are needed to investigate the biological mechanisms and the predicted attributable mortality for cold temperatures. Third, we used assumed levels of PM_10_ rather than climate predicting of PM_10_ levels through modeling. The prediction of PM levels has been performed by artificial neural networks [35], time-varying statistical models [36], and support vector machines [37] to overcome the difficulties in predicting the precipitation, wind, temperature, and relative humidity that affects PM_10_. In addition, we did not estimate the PM_10_ concentration where the relative risk considering only temperature is as much as the relative risk considering the interaction effect. However, our assumption is simple and easy to interpret. Also, the identical temperature-mortality relationship of the baseline in the projected periods was used, although different thresholds of each period were applied. Finally, this study included the limitation of ecological studies such as uncontrolled confounding and measurement error.

Overall, we found an increased modification on association between particulate matter and temperature-attributable deaths and identified higher risks for PM_10_ concentrations over 65 μg/m^3^. Our findings also showed substantial geographical variation in heavily populated areas, which is a point of increasing interest in worldwide integrated research. Therefore, this research provides an essential foundation for researchers and policy makers in understanding heat-attributable health burdens considering PM_10_.

## Figures and Tables

**Table 1:** The 95th and 99th percentile temperatures for the 229 districts in South Korea at baseline and in the projected periods (2030s, 2050s, and 2080s).

Percentile	RCP Scenario	Period			
		Baseline	2030s	2050s	2080s
95%	4.5	26.61 ± 1.14	26.74 ± 1.17	27.27 ± 1.25	28.02 ± 1.20
	8.5		26.75 ± 1.11	28.52 ± 1.29	29.83 ± 1.21
99%	4.5	28.46 ± 1.10	28.96 ± 1.25	29.49 ± 1.15	30.34 ± 1.16
	8.5		28.75 ± 1.15	30.80 ± 1.29	31.90 ± 1.25

**Table 2:** Ten-year-average attributable deaths and the 95% confidence interval of the baseline and projected periods (2030s, 2050s, and 2080s) by considering the relative risks of 229 districts.

Period/ Scenario		Levels of PM_10_ (in μg/m^3^)					
		0	35	50	65	80	95
Baseline		65.68 ± 62.7	51.53 ± 48.29	61.92 ± 59.88	68.72 ± 67.75	72.69 ± 73.19	74.89 ± 77.18
4.5	2030s	153.37 ± 160.84	120.34 ± 125.26	143.27 ± 151.94	161.9 ± 173.51	172.92 ± 186.53	179.97 ± 196.53
	2050s	366.2 ± 323.02	283.65 ± 247.46	342.62 ± 303.27	389.85 ± 346.93	417.03 ± 374.11	437.54 ± 395.21
	2080s	453.61 ± 383.98	351.81 ± 298.45	428.68 ± 365.06	488.63 ± 417.48	525.34 ± 449.94	552.62 ± 474.18
8.5	2030s	127.59 ± 124.56	100.07 ± 96.51	119.67 ± 118.3	135.05 ± 135.29	143.44 ± 145.29	149.1 ± 152.12
	2050s	540.54 ± 462.33	419.15 ± 357.01	512.19 ± 439.14	584.02 ± 501.57	628.69 ± 540.72	663.11 ± 570.31
	2080s	545.09 ± 428.93	422.34 ± 333.63	517.94 ± 408.48	591.23 ± 464.97	637.42 ± 501.01	673.91 ± 530.28

## Supplementary Materials

The following are available online at https://www.mdpi.com/1660-4601/17/7/2600/s1, Figure S1: The 95th and 99th percentile temperatures (°C) in the 2030s, 2050s, and 2080s, Figure S2: Association between temperature and mortality modified by air pollutants in 7 major cities in Korea, Figure S3: Association between lag on temperature and relative risk in considering various PM10, Figure S4: Ten-year-average attributable deaths and the 95% confidence interval for each period by considering the relative risks of 229 districts, Table S1: Mean relative risk and 95% confidence interval with various PM10 levels in the 229 considered districts.
