# Supplementary material for: Predicted Future Mortality Attributed to Increases in Temperature and PM10 Concentration under Representative Concentration Pathway Scenarios
Source: Int J Environ Res Public Health. 2020 Apr 10;17(7):2600. doi: 10.3390/ijerph17072600 (PMC7177966; doi:10.3390/ijerph17072600)
Supplement: Supplementary file 1 [file ijerph-17-02600-s001.pdf]

International Journal of Environmental Research and Public Health

MDPI

Supplementary Information for

Predicted future mortality attributed to increases in temperature and PM_{10} concentration under representative concentration pathway scenarios

Jiyun Jung[1], Jae Young Lee[2], Hyewon Lee[2, 3], Ho Kim[1, 2,*]

[1] Graduate School of Public Health, Seoul National University, 1 Gwanak-Ro, Gwanak-Gu, Seoul 08826, South Korea; bestjudy@hanmail.net

[2] Institute of Health and Environment, Seoul National University, 1 Gwanak-Ro, Gwanak-Gu, Seoul 08826, South Korea; jaeyoung.lee@alumni.stanford.edu

[3] Department of Neuropsychiatry, Seoul National University Bundang Hospital, Seongnam 13620, South Korea; woniggo@gmail.com

*Correspondence: hokim@snu.ac.kr; Tel.: +(82) 2 880-2701-3; Fax.: +82-(2)-745-9104.

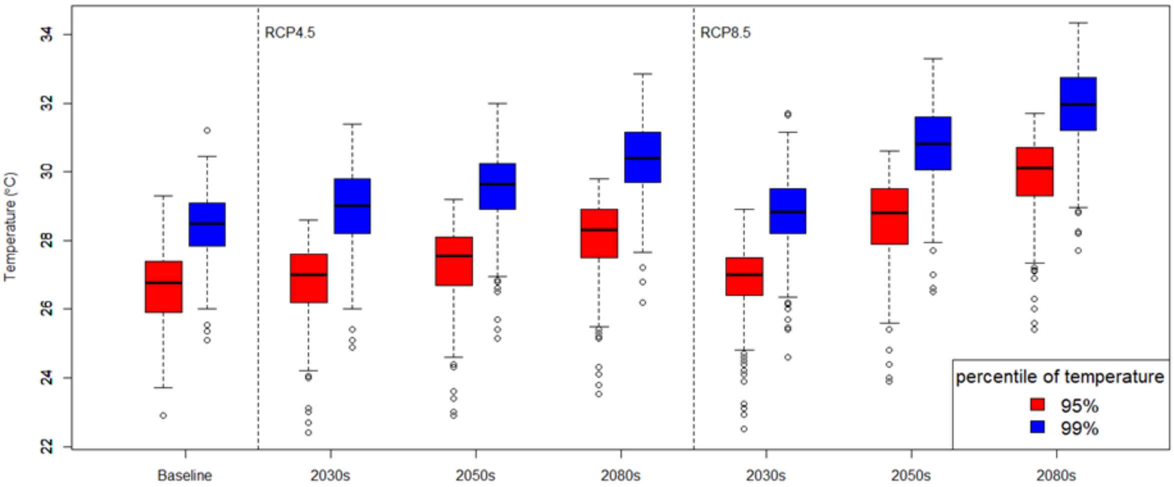

Figure S1. The 95th and 99th percentile temperatures (°C) in the 2030s, 2050s, and 2080s.

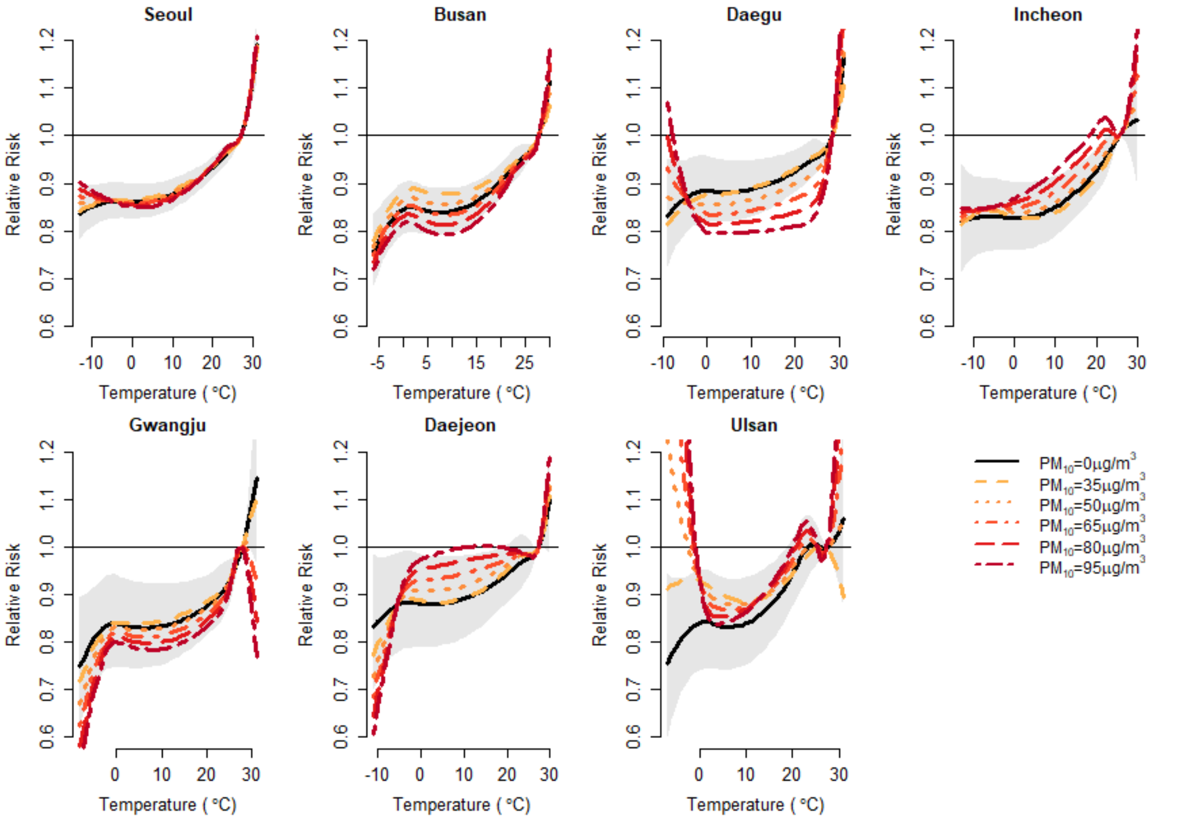

Figure S2. Association between temperature and mortality modified by air pollutants in 7 major cities in Korea.

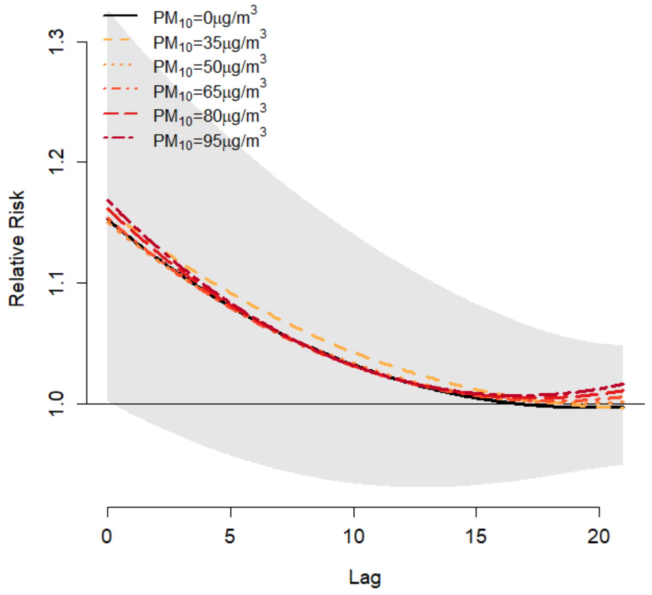

Figure S3. Association between lag on temperature and relative risk in considering various PM10.

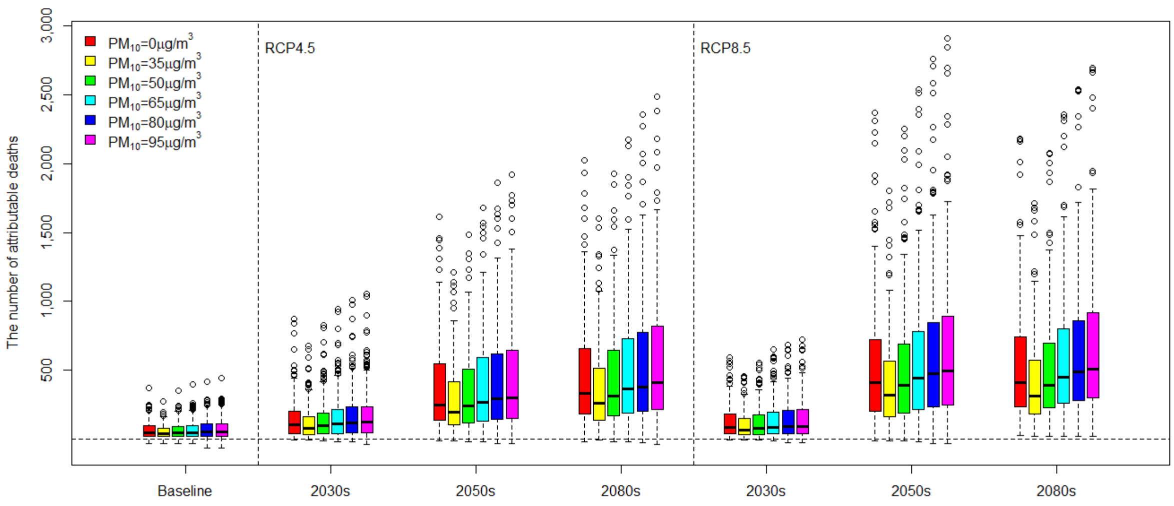

Figure S4. Ten-year-average attributable deaths and the 95% confidence interval for each period by considering the relative risks of 229 districts.

Table S1. Mean relative risk and 95% confidence interval with various PM10 levels in the 229 considered districts.

RCP scenarios/ Projection period		Levels of PM_{10} (in μg/m^3)					
		0	35	50	65	80	95
Baseline		1.07±0.05	1.05±0.03	1.06±0.04	1.07±0.05	1.08±0.06	1.08±0.06
RCP4.5	2030s	1.11±0.07	1.08±0.05	1.1±0.07	1.11±0.08	1.12±0.09	1.13±0.1
	2050s	1.13±0.07	1.1±0.05	1.12±0.07	1.14±0.08	1.15±0.09	1.16±0.09
	2080s	1.18±0.08	1.14±0.06	1.17±0.08	1.2±0.09	1.22±0.1	1.23±0.11
RCP8.5	2030s	1.09±0.06	1.07±0.04	1.08±0.06	1.09±0.07	1.1±0.07	1.1±0.08
	2050s	1.21±0.09	1.15±0.06	1.19±0.08	1.23±0.1	1.25±0.11	1.27±0.12
	2080s	1.24±0.08	1.17±0.05	1.22±0.07	1.27±0.09	1.29±0.1	1.31±0.11
